# The underestimated role of myopia in uncorrectable visual impairment in the United States

**DOI:** 10.1038/s41598-023-42108-y

**Published:** 2023-09-15

**Authors:** Mark A. Bullimore, Noel A. Brennan

**Affiliations:** 1 College of Optometry, University of Houston, Houston, TX USA; 2 Johnson and Johnson Vision, Jacksonville, FL USA

**Keywords:** Eye diseases, Refractive errors

## Abstract

We estimate the US prevalence of uncorrectable visual impairment in 2050 accounting for the changing distribution of both age and myopia. Age projections of the US population (from an estimated total of 379 million in 2050), were taken from the US census website. The distribution of myopia, by severity, was calculated from literature-derived prevalence estimates of 58.4% (≤ − 0.50 D, 2050 projection) and 33.1% (≤ − 1.00 D, 1999–2004 estimate) to provide predicted and conservative estimates, respectively. Uncorrectable visual impairment as a function of age and refractive error was modelled by multiple linear regression. Finally, the likely number of individuals in the US with visual impairment in 2050 was calculated. For a projected myopia prevalence of 58.4%, 222 million are projected to be myopic and 48 million will have high myopia (− 5 D or worse). The projected total number with uncorrectable visual impairment is 11.4 million of which 4.9 million cases (43%) of visual impairment will be directly attributed to increased risk of eye disease associated with myopia. For a projected myopia prevalence of 33.1%, 8.9 million are projected to have uncorrectable visual impairment of which 2.4 million cases (27%) will be directly attributed to myopia. It is predicted that between 27 and 43% of uncorrectable visual impairment in the US population in 2050 will be directly attributable to myopia. Failure to account for the increasing prevalence of myopia among the aging population leads to a substantial underestimate of the prevalence of visual impairment.

## Introduction

The eyecare community’s perspective on myopia has evolved from regarding it as a benign refractive condition to a fuller understanding of its role in eye disease and visual impairment^1^. Myopia increases the risk of posterior subcapsular cataract, glaucoma, retinal detachment, and most importantly, myopic maculopathy (myopic macular degeneration)^2^. Furthermore, the risk of these conditions increases with each diopter of myopia^3^. Myopia therefore increases a patient’s risk of uncorrectable (nonrefractive) visual impairment in later years^4^, with the risk again accumulating with each diopter of myopia^3^. The management of the above diseases can be more challenging in higher levels of myopia and the outcomes are poorer.

Recent projections of uncorrectable visual impairment have not accounted for increasing prevalence of myopia, particularly in older individuals^5,6^. Here, we estimate the prevalence of uncorrectable visual impairment (20/40 or worse best corrected) in the US in 2050 accounting for the distribution of both age and myopia. The goal is to quantify the role that myopia plays in uncorrectable visual impairment, including the relative contributions of high myopia (− 6 D or worse) and low myopia.

## Results

**Figure 1:**
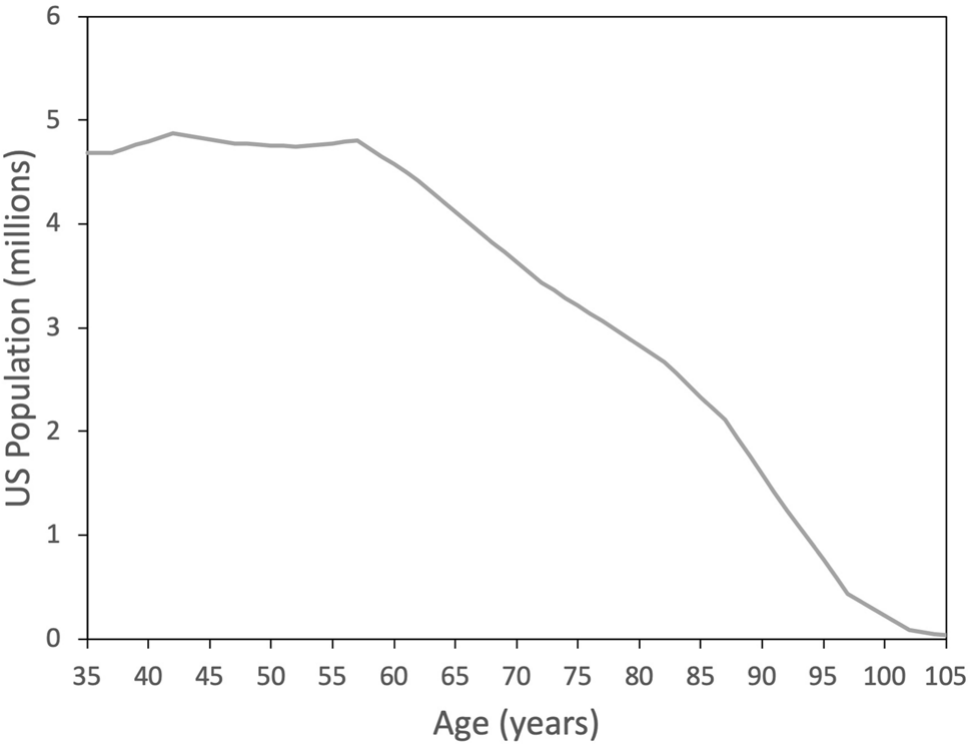
Projected age distribution for the US population in 2050 from the 2017 census.

**Figure 2:**
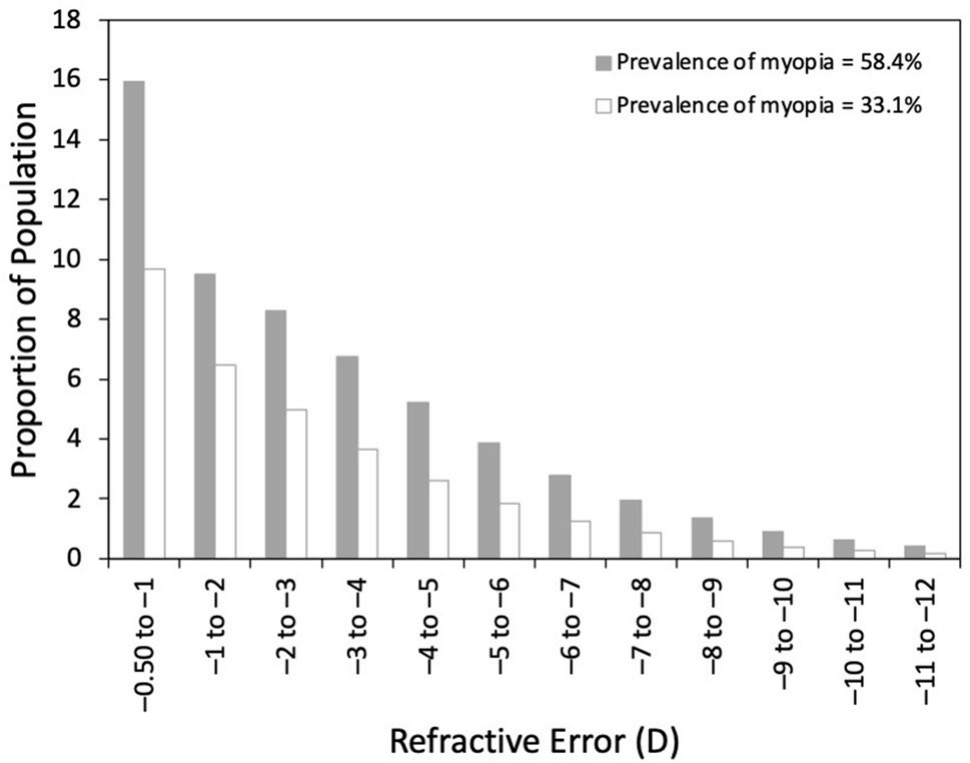
The estimated distribution of myopia by severity based on overall prevalence values of 58.4% and 33.1% for − 0.50 D or worse.^8^

Figure 1 shows the projected age distribution for the US population in 2050 based on US census data. Given the age-dependence of visual impairment and for consistency with previous estimates^6,7^, data are only shown for 35 years and above, but the distribution is relatively flat at younger ages. Figure 2 shows the estimated distribution of myopia by severity based on overall prevalence of 58.4% and 33.1% for − 0.50 D or worse^8^. The peak at − 0.50 to − 0.99 reflects the leptokurtotic distribution. Beyond –5 D, each diopter bin is around 70% of the previous^9^. It should be noted that for prevalence values greater than 80%, the distribution changes such that the peak of the distribution is now beyond − 2 D, but this has limited relevance to the US population^10,11^.

**Figure 3:**
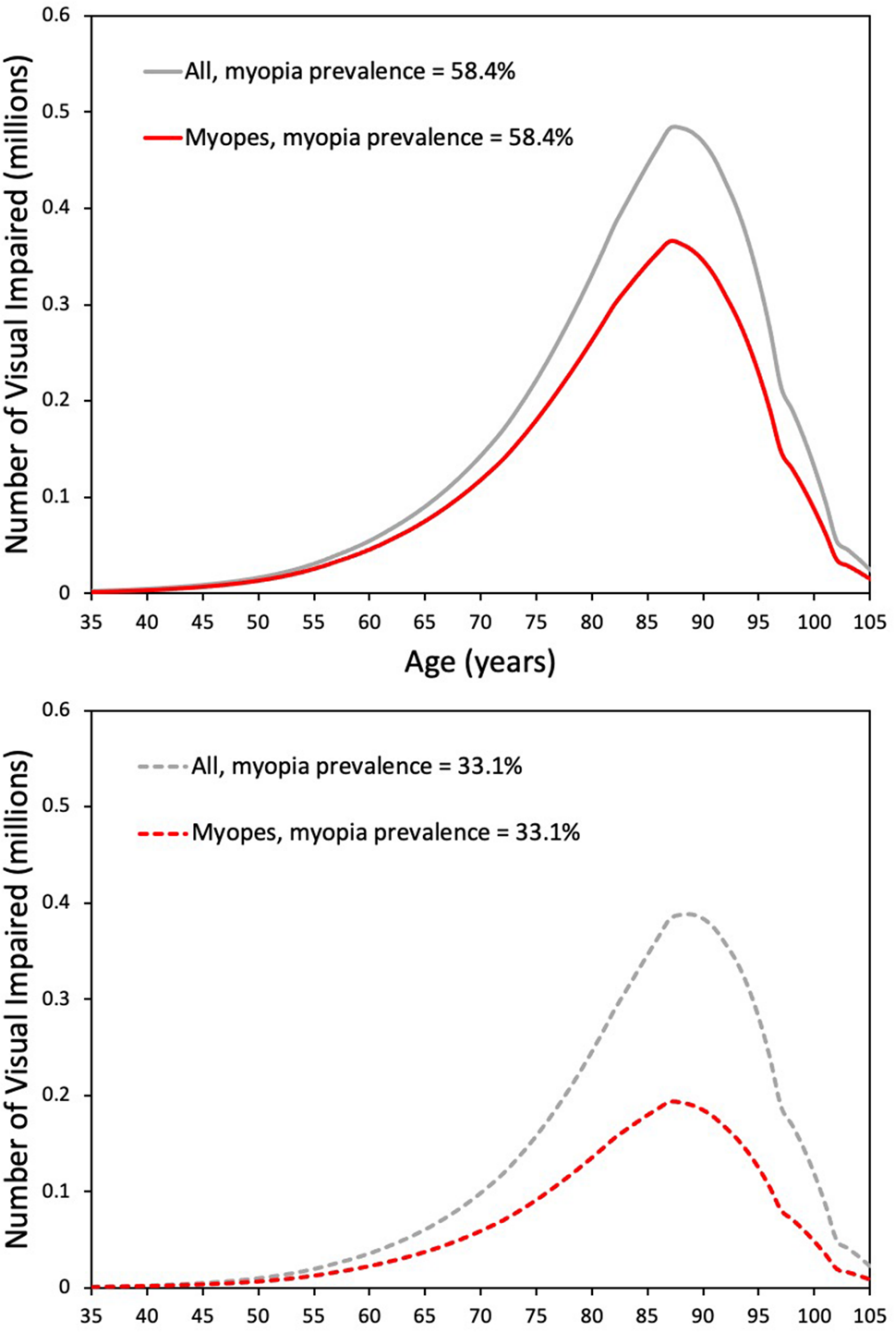
Predicted age distribution of visually impaired individuals (20/40 or worse best corrected) in 2050 based on overall myopia prevalence values of 58.4% and 33.1%.

Figure 3 shows the predicted age distribution of individuals with uncorrectable visual impairment (20/40 or worse best corrected) for myopia prevalences of 58.4% and 33.1%. Between 40 and 80 years the number of visually impaired increases exponentially with age, but thereafter mortality exerts a greater influence. Also shown is the age distribution for visually impaired myopes. For a myopia prevalence of 58.4%, the total number with visual impairment is projected to be 11.4 million of whom 8.7 million will be myopic. For a myopia prevalence of 33.1%, the total number with visually impairment is projected to be 8.9 million of whom 4.5 million will be myopic. It is apparent from these numbers and Fig. 3 that myopes are over-represented among the visually impaired. Thus, 76% and 51%, respectively, of the visually impaired are predicted to be myopic for the two prevalence estimates.

**Figure 4:**
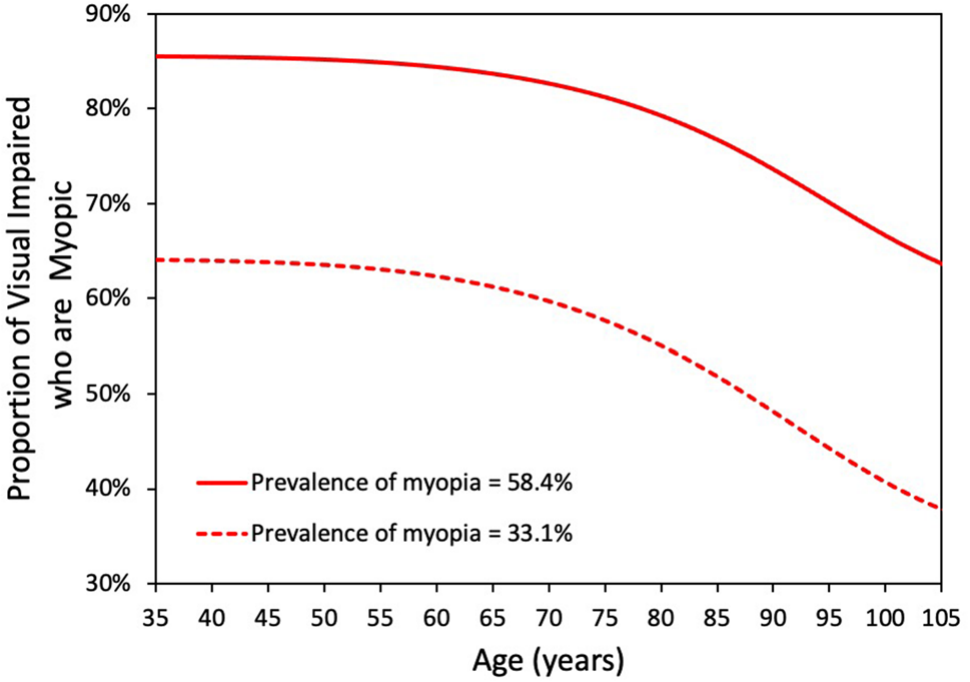
The proportion of predicted visually impaired individuals (20/40 or worse best corrected) in 2050 visually impaired who are myopic based on overall myopia prevalence values of 58.4% and 33.1% as a function of age. If myopia had no influence on visual impairment, the line would be horizontal.

Figure 4 shows the proportion of visually impaired who would be myopic for the two prevalence estimates as a function of age. If myopia had no influence on visual impairment, the lines would be relatively flat. It is apparent from these numbers and Fig. 3 that myopes are over-represented among the visually impaired. The proportions are, however, very much dependent on age. While less than 10% of the visually impaired are younger than 65 years, myopes account for a disproportionate number of these individuals, presumably because myopia-related eye diseases, principally myopic maculopathy, have earlier onset than many age-related conditions leading to visual impairment, such as age-related macular degeneration.

**Figure 5:**
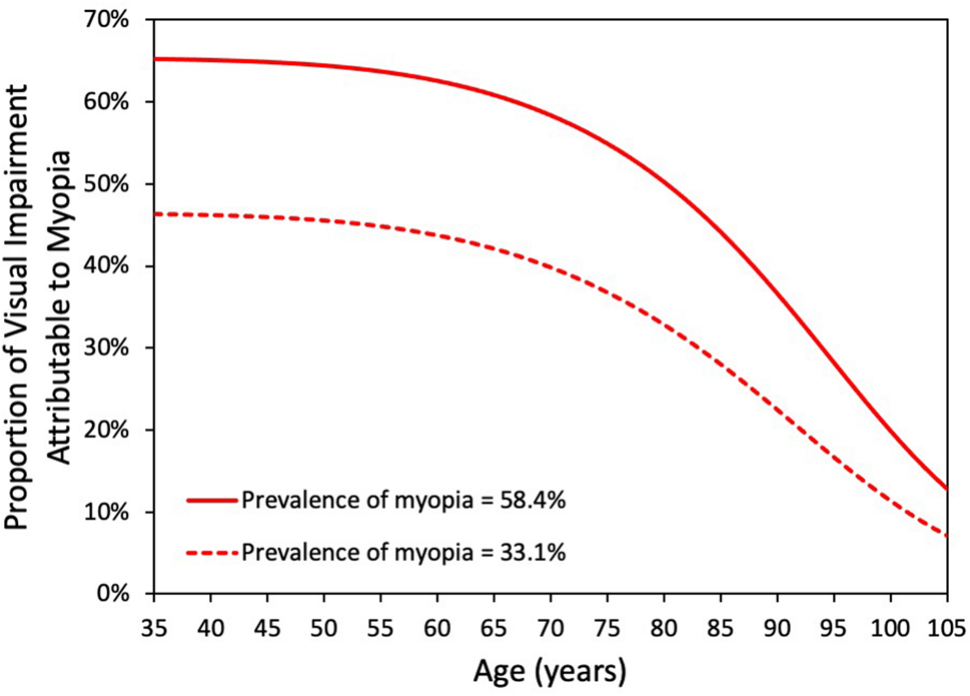
The proportion of predicted uncorrectable visual impairment in 2050 attributable to myopia based on overall myopia prevalence values of 58.4% and 33.1% as a function of age. Were myopia not a risk factor for visual impairment, the line would be horizontal at 0%.

The role of myopia in visual impairment is further demonstrated in Fig. 5 which shows the proportion attributable to myopia as a function of age. Were myopia not a risk factor for visual impairment, the value would be 0% at all ages. Were the entire population non-myopic, the model predicts 6.5 million visually impaired compared with 11.4 million for a projected myopia prevalence of 58.4%, meaning that myopia would be responsible for 43% of visual impairment (4.9 million). If only 33.1% of the population is myopic in 2050, myopia would be responsible for 27% of visual impairment (2.4 million). Again, these estimates are highly age-dependent with myopia accounting for over 60% of visual impairment among working age adults for a projected myopia prevalence of 58.4%. Even at 80 years, 50% of visual impairment is attributable to myopia.

**Figure 6:**
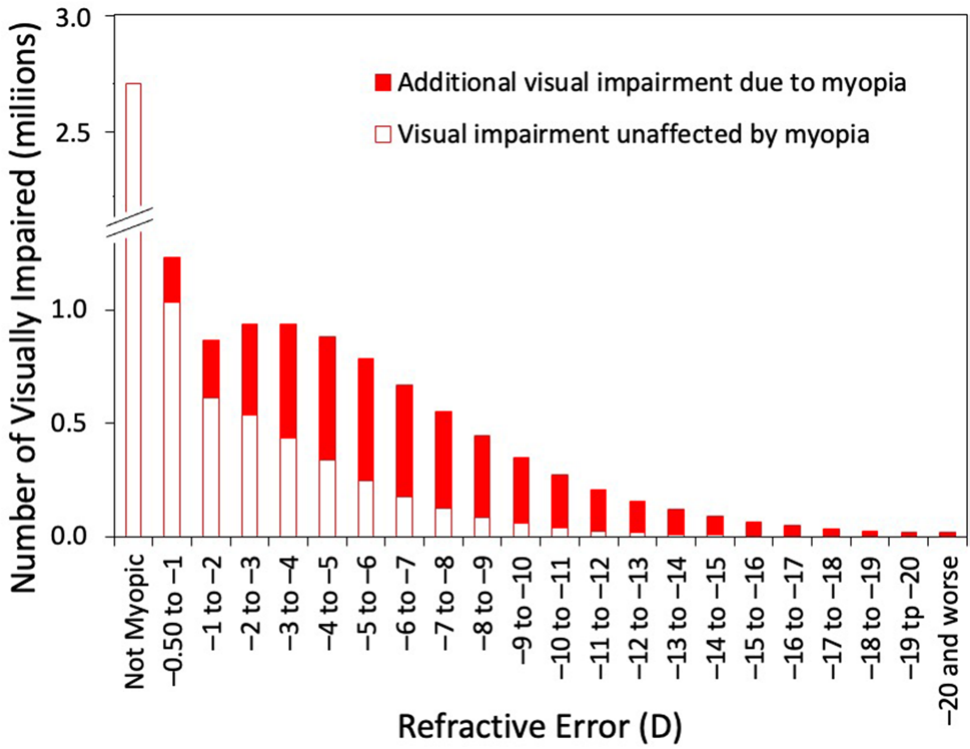
The predicted distribution of uncorrectable visual impairment in 2050 as a function of myopia level. The red portion of the bars represent the visual impairment attributable to myopia.

Figure 6 shows the predicted distributions of visual impairment, both due to and independent of myopia, as a function of myopia level for a prevalence of 58.4%. The number of visually impaired myopes is relatively constant from − 1 to − 5 D, as the increased risk of visual impairment with increasing levels of myopia is offset by the decrease in the overall number of myopes. Only 3.1 million of the visually impaired are myopes − 6 D or worse, compared with 5.6 million myopes between − 0.50 and − 6 D and 2.7 million non-myopes. Of the 4.9 million whose visual impairment is attributable to myopia, it is estimated that 2.4 million (49%) would be low or moderate myopes, emphasizing that the impact of myopia is not restricted to high myopia.

Table 1 expands on the influence of myopia prevalence on estimates of visual impairment (20/40 or worse best corrected) and the amount attributable to myopia. Several features are worthy of note. First, the established relation between myopia and visual impairment can be articulated further: each 10% increase in the prevalence of myopia increases the number of visually impaired by about 1 million. Second, the visual impairment attributable to myopia increases with the underlying prevalence from 2.4 million at 33.1% to 5.1 million at 60%. Finally, the effect of controlling myopia by 1 D can also be estimated, essentially by shifting the distribution in Fig. 2 to the left by one bin. The result is that, for an underlying prevalence of myopia of 58.4%, the predicted visual impairment is reduced by 2.0 million (14%). Of course, this could only be achieved by treating every myope and also preventing the onset of myopia in a significant number of individuals^12^. It is more practical to consider the impact of treating children destined to be above a certain level of myopia. For example, treating all children who will, left untreated, progress to − 6 D or worse, would theoretically lower visual impairment by 0.6 million (5%). Adjusting the criterion to − 3 D or worse would lower visual impairment by 1.3 million (11%), but of course, many more children would need to be treated to attain such a goal.

**Table 1:** The predicted number (in millions) of visually impaired (20/40 or worse best corrected) in the US in 2050 as a function of the underlying prevalence of myopia.

Prevalence of myopia (%)	Number of visually impaired	Visual impairment attributable to myopia	Visual impairment prevented by 1 D control
33.1	8.9	2.4 (27%)	1.0 (12%)
40	9.5	3.0 (32%)	1.3 (13%)
50	10.4	3.9 (38%)	1.6 (15%)
58.4	11.4	4.9 (43%)	2.0 (17%)
60	11.6	5.1 (44%)	2.0 (17%)

The number of visually impaired attributable to myopia and the number that could be prevented by 1 D control are also shown.

## Discussion

This study represents the first modelling of future visual impairment in the US accounting for the ongoing and projected increase in myopia. Using our published relationship of visual impairment, age, and myopia, we demonstrate that myopia will account for around a third of uncorrectable visual impairment. We also show that each 10% increase in the prevalence of myopia could result in another million cases of visual impairment. Thus, efforts to prevent the onset of myopia and slow its progression should have a profound public health benefit. Our primary estimates are based on the projected 2050 prevalence of myopia from Holden et al.^8^ We also use an estimate of myopia prevalence (33.1%) that is conservative^13^ in multiple ways: (i) it applies a more stringent criterion of − 1.00 D; (ii) it is 2 decades old (1999–2004 NHANES data), and (iii) it assumes no future increase. Even with this more conservative approach, the analysis shows that myopia is, and will continue to be, responsible for a significant proportion of uncorrectable visual impairment in the US.

Table 1 shows how the projected number of individuals with uncorrectable visual impairment varies with the prevalence of myopia. For example, a 20% increase in myopia prevalence from 40 to 60% results in 2.1 million additional visually impaired individuals. The influence of age on the prevalence of visual impairment is well established^5–7^, and the exponential relationship used here has been validated previously^6^. Thus, as the US population ages, visual impairment will rise. The quality of our model can be assessed by comparison to other analyses. For example, applying our model to the 2000 US population of 282 million and a prevalence of myopia of 33.1%^13^ yields an estimate of 2.8 million visually impaired aged 40 years or older, consistent with the 3.3 million reported by the Eye Diseases Prevalence Research Group^7^. Of the estimated 2.8 million, the model predicts that 0.87 million (31%) cases are attributable to myopia. As shown in Table 1, applying the same prevalence of myopia to the 2050 US population of 379 million yields an estimate of 8.9 million visually impaired. Thus, while the population is estimated to increase by 34% in the first half of this century, the number of visually impaired is projected to triple. This is because most of the population growth will occur in individuals older than 65 years.

This analysis emphasizes the likely contribution of low and moderate myopia to future visual impairment. Just under 50% of predicted visual impairment that is solely attributable to myopia will occur in patients with less than − 6 D of myopia. This is consistent with reports of a similar proportion of subjects with myopic maculopathy having low or moderate myopia^14–17^ and is a function of both the lower, but significant, risk of sight-threatening diseases at low and moderate levels of myopia^2^, but the much greater frequency of these degrees of refractive error. Perkins noted that risk of retinal detachment and glaucoma increases even at low levels of myopia and Flitcroft reiterated that “there is no safe level of myopia”^18^. The American Academy of Ophthalmology Taskforce on Myopia also noted that “although higher degrees of myopia carry a greater risk of visual impairment to the individual, the population-based burden of lower degrees of myopia remains considerable”^1^. The implication is that treatment to reduce onset or progression at all levels of myopia, not just those prone to developing high myopia, is necessary to impact the overall public health burden from myopia.

The model quantifies the contribution of myopia to uncorrectable visual impairment. An increase in the US prevalence of myopia from the 33.1% reported by Vitale et al.^13^ at the turn of the century, to Holden et al.^8^ 2050 prediction of 58.4% results in an additional 2.5 million individuals with uncorrectable visual impairment (Table 1). This predicted increase in prevalence elevates the proportion of visual impairment attributable to myopia from 27 to 43%. Of course, the projections of Holden et al. for the US are based largely on the data of Vitale et al.^13^.

Varma et al.^5^ used data from six major US population-based studies of visual impairment to estimate the prevalence of uncorrectable visual impairment. They estimate that between 2015 and 2050 the prevalence will more than double from 3.22 to 6.95 million. The discrepancy with the current estimate of 11.4 million, based on a 2050 prevalence projection of 58.4%, is reasonably explained by our inclusion of the additional projected burden of myopia and reliance by Varma et al. on a number of studies published in the 1990s. Likewise, Chan et al. applied an established exponential model of age-related visual impairment and estimated that in 2050 there will be 7.6 million visually impaired adults over the age of 45 years.^6^ The authors use more recent visual acuity data from the 2007–2008 NHANES, with representation of racial and ethnic groups. Our conservative estimate of 8.9 million predicts a 20% increment over those data, potentially as a result of the impact of myopia.

Our estimates of visual impairment associated with myopia are consistent with those of Fricke et al.^19^ They estimated that in 2050, 55.7 million individuals worldwide will be visually impaired as a result of myopic maculopathy, which is the main, but not only, cause of visual impairment attributable to myopia. Scaling these numbers to the US population yields 2.3 million. Fricke et al. define visual impairment as 20/60 or worse, a criterion that reduces the prevalence of visual impairment by a factor of three^6,20^. Applying a proportionately lower underlying prevalence of 0.5% to our model predicts 5.3 million cases of visual impairment of which 2.7 million are attributable to myopia, but that includes all myopia-related disease.

If the estimates of Holden et al. for myopia prevalence are realized^8^, the complications of myopia will likely become the biggest cause of uncorrectable visual impairment in the United States in coming decades. This is a stunning development given that myopic maculopathy, arguably the most serious complication of myopia, has not been followed as a separate cause of visual impairment globally, even in recent surveys^21,22^. Holden et al. predicted that the prevalence of high myopia in east Asia and high income Asia–Pacific in 2020 was 13.8% and 10.1%, respectively. Already, “pathologic myopia is the leading cause of irreversible blindness in Taiwan, Japan and China”^23^. The projected prevalence of high myopia in North America in 2050 is 14.8%, above that currently estimated for these regions. Efforts to delay and slow progression in children today in 10-year-olds will only reap major benefit in 2070 and beyond, when these individuals turn 60. The large numbers of high myopes aged 20 years and above in some East Asian populations are destined to create the alarming scenario we describe here, unless improved treatments for the sight threatening conditions are developed. In the event that the projections of Holden et al.^8^ are not realized, our conservative estimates predict that myopia remains an important, previously underestimated, contributor to visual impairment.

In their seminal paper, Tideman et al.^4^ forecast 2- to threefold increases in visual impairment in Europe by 2055. Our model yields a similar prediction for the US. Tideman and colleagues also estimate that in South Korea more than 10% of the population aged 75 years or above will have visual impairment attributable to myopia. Our model predicts that in the US, 9.2 million of 48 million individuals in this age range will be visually impaired, of which 2.3 million (5%) will be attributable to myopia.

The predictions presented here do not address uncorrected refractive error and cataract, both of which contribute to correctable visual impairment. In the 1999–2002 NHANES sample, 1190 participants presented with visual impairment, but 83% could achieve 20/40 or better once corrected^24^. Thus, myopia is also a major contributor to correctable visual impairment. Cataract accounts for some mild visual impairment observed in studies of western populations^25^. Those studies reporting higher rates of visual impairment show lower rates of cataract surgery^26^. Tideman et al.^4^ excluded participants with cataract in their estimates of uncorrectable visual impairment. Early reports on one of their cohorts indicates that cataract is the cause of 30% of cases of visual impairment and blindness^20^. Cataract places a large burden on the ophthalmic community and patients. While routine, cataract surgery is not without complications and may increase the risk of age-related macular degeneration^27^. Finally, nuclear cataract may cause myopic shifts in refraction. The stronger association between axial length and visual impairment reported by Tideman et al. suggests this plays a minor role^4^.

### Limitations

The modelling described herein is highly dependent on the validity of the assumptions. There are limited data on the relation between refractive error and visual impairment and our model relies on data from the Netherlands—a largely non-Hispanic white population^4^. While the cohorts are well-described and include over 15,000 individuals with measures of both corrected visual acuity and refractive error, visual impairment in the US is around 50% higher in Hispanic whites and African Americans than in non-Hispanic whites. Likewise, myopia is less common among African Americans^13^. Thus, applying data from a more ethnically-diverse population would improve future estimates for the US population and for other regions. Nonetheless, the rates of visual impairment in the Netherlands^20^ are similar to those reported in some white US populations^25,28^ and lower than in others^29^. The Netherlands also has a well-developed public health system, potentially yielding better healthcare outcomes than in the United States. Therefore, application of the Tideman data may lead to an underestimation of the number of visually impaired in our projections, although the relation between age and visual impairment is similar in Dutch and white US populations^7^. Further, while the Netherlands cohorts include subjects up to 89 years, data were only presented through 75 years, so some extrapolation is needed. Given that our model predicts that over half of the visually impaired are above 80 years, due caution should be exercised. Of course, these older individuals may be underrepresented in population-based studies. Participation in major health surveys is lower among older individuals^24^ and rates of visual impairment among nursing home residents are 50% or higher^30,31^.

Our primary results rely on the projections of Holden et al.^8^ The data underlying these projections for the US are based on the NHANES data as analyzed by Vitale et al.^13,32^ Vitale and colleagues make a compelling case for the prevalence of myopia being significantly higher in 1999–2004 than in 1971–1972 (41.6% vs 25.0%, respectively) supporting an upward trajectory. Furthermore, the 1999–2004 prevalence of myopia in those 20–39 years and 40–59 years was estimated as 50.2% and 50.1%, respectively, using a criterion of − 0.50 D^13^. Applying the more conservative criterion of − 1.00 D, the corresponding values are 36.2% and 37.6%. In contrast, the prevalence in those 60 years and older is substantially lower for both criteria. The 95% confidence interval for all these values is around ± 2.5%.

It should be noted that the NHANES refractive error data were obtained without cycloplegia. This should have little impact on adults 40 years and older due to presbyopia. The influence of cycloplegia on objective refraction in younger adults is equivocal. Fotouhi et al. found a difference of around 0.4–0.5 D at ages between 16 and 50 years^33^. This would support the use of the − 1.00 D criterion for myopia if noncycloplegic refraction is used. Conversely, Sanfilippo et al. found that the mean difference between pre- and post-cycloplegic autorefraction decreased from 0.36 D in 13-year-olds to only 0.06 D in 25-year-olds^34^. Some authoritative sources have argued that cycloplegic refraction should be the gold standard for epidemiological studies of myopia^35^. While we agree, autorefractors may vary in the ability of their fogging mechanism to relax accommodation in adults and those with a larger entrance pupil may result in changes in refraction with cycloplegia due to ocular aberrations, rather than accoomodation^35,36^. The myopia prevalence estimates from NHANES^13^ are consistent with other values in older US adult populations. For example, the 1985–1988 Baltimore Eye Survey reported a myopia prevalence of 40.9% in white participants between 40 and 49 years^28^. Likewise, the 1987–1988 Beaver Dam Eye Study found a myopia prevalence of 42.9% in adults between 43 and 54 years of whom 99% were white^37^. Both values from the 1980s are lower than the 1999–2004 NHANES estimates in 40–60-year-olds but higher than the overall prevalence in 1971–1972 and none used a cycloplegic. Of course, whether the prevalence of myopia in the US will continue to increase through 2050 is open to discussion, hence a range of values are shown in Table 1.

The model assumes a constant prevalence across all ages. Inspection of the projections of Holden et al. for 2050 shows prevalence increasing from birth through age 20 years and relatively constant thereafter. Indeed, their global prevalence estimate of around 50% represents steadily increasing prevalence in childhood and is between 63 and 67% across all adult ages. Based on their data, applying a constant prevalence across the age range susceptible to visual impairment is appropriate, although applying a population-wide value may underestimate the projected prevalence in older adults by 5–10%. The prevalence of myopia in children and younger adults is under-researched in the US and further studies would refine the estimates used in our model. The systematic review by Rudnicka et al. only presents data for US 5-year-olds with no estimates of older US children^38^. Of course, ongoing myopia progression and new cases during adulthood^39^ mean that estimates in older adults are more important to understanding the full impact of myopia on visual impairment, although it is unknown whether a − 7 D myope who develops all that myopia during childhood has a different risk from one who developed half during childhood and the remainder as an adult. As discussed above, estimates of future prevalence in adults rely heavily on the NHANES data^13,32^, although other US-based studies support an increase over time^37,40^. Bomotti et al. reported a 10–15% higher prevalence of myopia among adults aged 43–74 years born between 1943 and 1947, compared to those born 10 years earlier^41^. Nonetheless, there is a need for more contemporary data.

The population mean refractive error shifts in a myopic direction during childhood as myopia develops in a portion of individuals and progresses thereafter. It can also progress into early adulthood^39^. Even once myopia has stabilized, refractive error will change later in adulthood. Lens changes in a patient’s fifties can result in hyperopic shifts and, later, nuclear opacity can cause myopic changes^42^. These lenticular changes can alter the refractive distribution of a population and thus estimates of the prevalence of myopia. Estimates of the relationship between myopia-associated eye disease and refractive error are currently based on refractions conducted on older age groups in whom these diseases are mostly found^4^. Assuming that any myopia-related increase in morbidity is a result of axial elongation, using prevalence estimates in earlier adulthood may be prudent to avoid confounding lenticular changes. Interestingly, the 20–40-year-olds who participated in NHANES between 1999 and 2004 will be between 70 and 90 years in 2050 and thus at greatest risk of visual impairment. Thus, a case could be made for focusing on the prevalence of myopia in this cohort (50.2% using a criterion of − 0.50 D or 36.2% for a criterion of − 1.00 D) when estimating future visual impairment (Table 1).

### Summary

Myopia is a major contributor to visual impairment and is predicted to become a major contributor in years ahead. Continued efforts to prevent myopia, delay its onset, and slow its progression should have a profound influence on future levels of visual impairment.

## Methods

The broad approach is to apply the risk of visual impairment as a function of age and myopia to the projected United States population in 2050. The model of Bullimore et al.^3^ is based on the comprehensive data of Tideman et al.^4^. The odds of visual impairment were calculated using a reference prevalence of 1.26% and multiple linear regression was used to estimate log_10_ odds as a function of age and refractive error (Rx). This result can be described as

$${\text{Odds of Visual Impairment }} = { 1}0^{{(0.0{\text{57Age }}{-} 0.{\text{122Rx }}{-}{ 4}.0{3})}}$$

Note that the visual impairment data underlying the model are from the Netherlands and now 10 years old but are agnostic with respect to cause of visual impairment. In other words, the dataset incorporates the risk of diseases that are and are not associated with myopia, e.g., age-related macular degeneration. The prevalence of visual impairment as a function of age and myopia level may then be calculated based on the underlying prevalence. Here we use the value of 1.4% for the prevalence of visual impairment (20/40 or worse) from the 1999–2002 US National Health and Nutrition Examination Survey (NHANES)^43^ as this represents a comprehensive dataset and myopia prevalence data are available from the same study^13^.

To apply the above model, estimates of the distribution of age and refractive error are needed. The projected age distribution of the US population was taken from the US census website (https://www.census.gov/data/datasets/2017/demo/popproj/2017-popproj.html). The overall prevalence of myopia (− 0.50 D or worse) was taken from Holden et al.^8^ From this overall value of 58.4%, the distribution of myopia by severity was determined using a universal calculator^9^. This calculator takes the overall prevalence of myopia for any criteria, e.g. − 0.50 D, and determines the prevalence in 1-D steps. We also use a range of estimates of prevalence, notably the more conservative 1999–2004 estimate of 33.1% in US adults above 20 years of age from NHANES, based on a criteria of − 1.00 D by non-cycloplegic refraction^13^.

The distributions of myopia and age were then convolved with cumulative risk of visual impairment to determine the number of visually impaired as a function of age and myopia. The proportion of visually impaired who are myopic was determined as a function of age. Finally, the amount of visual impairment attributable to myopia was estimated by modelling the age distribution of visual impairment if the entire population was non-myopic. Results are presented for different values for the prevalence of myopia between 30 and 60%.

The University of Houston Institutional Review Board determined that this research did not involve human subjects and thus review and approval was not required.

## Data Availability

The datasets analyzed in this work are publicly available.
